# Diversity of haemosporidian parasites in cranes: description of *Haemoproteus balearicae* and its phylogenetic position within the *H. antigonis* clade

**DOI:** 10.1051/parasite/2025059

**Published:** 2025-10-13

**Authors:** Mamohale Chaisi, Ndzalama Mabunda, Delphine Gey, Nkitseng Oageng Modise, Marli de Bruyn, Alexis Lécu, Sylvie Laidebeure, Anaïs Saillier, Milan Thorel, Tatjana Iezhova, Gediminas Valkiūnas, Monica Mwale, Linda Duval

**Affiliations:** 1 Foundational Biodiversity Science, South African National Biodiversity Institute P.O. Box 754 Pretoria 0001 South Africa; 2 Department of Veterinary Tropical Diseases, University of Pretoria Onderstepoort 0110 South Africa; 3 Département Adaptations du Vivant (AVIV), Molécules de Communication et Adaptation des Microorganismes (MCAM, UMR 7245 CNRS), Muséum National d’Histoire Naturelle, CNRS, CP 52 57 rue Cuvier 75231 Cedex 05 Paris France; 4 Parc zoologique de Paris, Muséum National d’Histoire Naturelle 53 Avenue de Saint-Maurice 75012 Paris France; 5 La Ménagerie, le zoo du Jardin des Plantes, Muséum National d’Histoire Naturelle Paris France; 6 State Scientific Research Institute Nature Research Centre Akademijos 2 08412 Vilnius Lithuania; 7 National Institute for Theoretical and Computational Sciences Durban KwaZulu-Natal South Africa

**Keywords:** Gruidae, *Haemoproteus*, *Cytb* gene, Birds, Phylogenetic clade

## Abstract

Haemosporidian parasites from the genera *Haemoproteus*, *Plasmodium*, and *Leucocytozoon* are significant avian pathogens. This study aimed to identify and characterize these parasites in cranes (family Gruidae), using combined morphological and molecular methods. The results confirmed the presence of *Haemoproteus balearicae*, redescribed here from *Balearica regulorum* and associated with *cytb* lineage hBAREGI210. This lineage, previously assigned to *Haemoproteus antigonis*, is reassigned to *H. balearicae*, suggesting possible cryptic speciation within the *H. antigonis* complex. The findings broaden the known host range and geographic distribution of *H. balearicae*, detected in captive-born cranes in France and captive cranes housed in conservation facilities in South Africa. Phylogenetic analyses revealed three distinct *Haemoproteus* clades in Gruidae, corresponding to at least three species, including *H. balearicae* and lineages representing *H. antigonis*. These crane-specific parasites may require taxonomic revision as a separate subgenus or genus, pending further studies on their life cycles and vectors. Additionally, several novel *cytb* lineages of *Plasmodium* and *Leucocytozoon* were detected, many unassigned to morphospecies. Notably, the pCATUS05 lineage, a member of the *Plasmodium lutzi* group previously reported only in the Americas, was detected for the first time in South African cranes, along with *Leucocytozoon* aff. *californicus* (lCIAE02), a widespread lineage lacking morphological description. Together, these findings reveal underestimated genetic diversity of haemosporidian parasites in cranes and highlight the importance of combining morphological and molecular data to clarify parasite taxonomy and host associations. This study advances our understanding of avian parasite ecology and systematics, with implications for crane conservation and disease management.

## Abstract (fr)

Les parasites hémosporidiens des genres *Haemoproteus*, *Plasmodium* et *Leucocytozoon* sont d’importants agents pathogènes aviaires. Cette étude visait à identifier et à caractériser ces parasites chez les grues (famille Gruidae), en utilisant des méthodes morphologiques et moléculaires combinées. Les résultats ont confirmé la présence d’*Haemoproteus balearicae*, redécrit ici chez *Balearica regulorum* et associé à la lignée *cytb* hBAREGI210. Cette lignée, précédemment attribuée à *Haemoproteus antigonis*, est réattribuée à *H. balearicae*, suggérant une possible spéciation cryptique au sein du complexe *H. antigonis*. Ces résultats élargissent la gamme d’hôtes et la répartition géographique connues de *H. balearicae*, détecté chez des grues nées en captivité en France et des grues captives hébergées dans des centres de conservation en Afrique du Sud. Les analyses phylogénétiques ont révélé trois clades distincts d’*Haemoproteus* chez les Gruidae, correspondant à au moins trois espèces, dont *H. balearicae* et des lignées représentant *H. antigonis*. Ces parasites spécifiques aux grues pourraient nécessiter une révision taxonomique en tant que sous-genre ou genre distinct, dans l’attente d’études complémentaires sur leurs cycles biologiques et leurs vecteurs. De plus, plusieurs nouvelles lignées *cytb* de *Plasmodium* et *Leucocytozoon* ont été détectées, dont beaucoup n’étaient pas attribuées à des morphoespèces. Notamment, la lignée pCATUS05, un membre du groupe *Plasmodium lutzi* précédemment signalé uniquement dans les Amériques, a été détectée pour la première fois chez des grues sud-africaines, ainsi que *Leucocytozoon* aff. *californicus* (lCIAE02), une lignée répandue manquant de description morphologique. Ensemble, ces résultats révèlent une sous-estimation de la diversité génétique des parasites hémosporidiens chez les grues et soulignent l’importance de combiner les données morphologiques et moléculaires pour clarifier la taxonomie des parasites et les associations avec les hôtes. Cette étude améliore notre compréhension de l’écologie et de la systématique des parasites aviaires, avec des implications pour la conservation des grues et la gestion des maladies.

## Introduction

Cranes (family Gruidae, order Gruiformes) are globally distributed and classified into two subfamilies, Crowned cranes (Balearicinae) including the Black Crowned crane (*Balearica pavonina*) and the Grey Crowned crane (*Balearica regulorum*), and the typical cranes (Gruinae) which comprise 13 species, classified into five genera: *Anthropoides, Antigone*, *Bugeranus, Grus*, and *Leucogeranus* [66]. The populations of crane species are declining due to habitat loss, land-use changes, hunting, illegal trade, and climate change [4]. As wetland indicators and culturally symbolic species [22], cranes are the focus of global conservation efforts, including both *in situ* and *ex situ* programs [42].

Haemosporidian parasites (order Haemosporida, phylum Apicomplexa) are widespread blood and tissue parasites transmitted by blood-sucking dipteran insects [61]. They are highly diverse and infect birds of most avian families [17, 56]. Over 260 species of the three main genera, *Plasmodium*, *Haemoproteus*, and *Leucocytozoon*, have been described worldwide [23, 34]. *Haemoproteus* and *Plasmodium* cause haemoproteosis and avian malaria, respectively, while *Leucocytozoon* infection leads to leucocytozoonosis [14, 27, 63]. These infections can threaten wild and captive bird populations by impacting survival and reproduction [13, 36, 49, 52, 61]. Their effects range from asymptomatic to lethal, particularly through anaemia, and have contributed to bird declines in Hawaii, New Zealand, the Galapagos, and in zoological parks [1, 12, 18, 39, 44], including mortality in cranes [37].

Four haemosporidian parasites have been identified in cranes, namely, *Haemoproteus antigonis, H. balearicae*, *Plasmodium polare*–like, and *Leucocytozoon grusi* [5, 6, 20, 60]. *Haemoproteus antigonis* was first described by deMello in 1935 from wild Demoiselle cranes (*Anthropoides virgo*) in India and later identified in Sandhill cranes (*Grus canadensis*) [25]. A redescription of *H. antigonis* from Sandhill crane blood smears was proposed by Bennett *et al.* [6] due to the lack of the type material in the original parasite description. More recently, *H. antigonis* has been reported in several other crane species, including *Grus americana*, *A. paradiseus*, and *B. regulorum* [10, 57, 58]. Phylogenetic analyses based on cytochrome *b* sequences suggest that this parasite forms a distinct and novel clade within *Haemoproteus* [10, 26]. *Haemoproteus balearicae* was first described from captive *B. pavonina* imported from the Democratic Republic of the Congo [50]. This parasite may also infect other Gruidae such as *B. regulorum* and *B. carunculatus* [61]. However, no recent morphological descriptions or *cytb* sequences are available for this species. A *Plasmodium polare*–like parasite was described in Sandhill cranes from Florida [60], and *Leucocytozoon grusi* was first reported in the same host in 1974 [5]. Recent studies have revealed a high diversity of *Plasmodium* in cranes, including *P. relictum*, *P. homonucleophilum*, and several unidentified *cytb* lineages, indicating their susceptibility to multiple malaria parasites [3, 9, 37]. *Cytb* lineages related to *Leucocytozoon majoris* and other unidentified *Leucocytozoon* parasites have also been detected in cranes [9, 37, 58].

Institutions such as the Parc Zoologique de Paris, the Jardin des Plantes Zoo (MNHN), and the South African National Biodiversity Institute (SANBI) contribute to the conservation of threatened birds, including cranes, through captive breeding programs and disease monitoring. Endangered crane species, including *B. regulorum*, *B. pavonina*, *A. paradiseus*, *A. virgo*, and *B. carunculatus* [32], are maintained in zoos and captive breeding facilities in France and South Africa. However, studies on haemosporidian infections in Gruidae remain scarce [9, 10, 37, 57, 67], with only one report from Africa [58].

This study aimed to investigate *Haemoproteus* parasites and other Haemosporida species, in five captive crane species in France and South Africa, where data are lacking. By combining morphological, molecular, and phylogenetic approaches, the study aimed to provide a comprehensive understanding of the taxonomic diversity of parasites infecting cranes and to clarify the phylogenetic relationships between *Haemoproteus* parasites in cranes and other haemosporidian parasites.

## Material and methods

### Ethics statement

In France, blood samples were collected exclusively for routine veterinary health monitoring and diagnostic purposes by accredited zoo veterinarians at the Parc Zoologique de Paris and La Ménagerie du Jardin des Plantes (MNHN). No animals were handled or sampled specifically for this research. Only a surplus portion of each blood sample was used in this study, in accordance with French and European ethical guidelines governing the secondary use of veterinary samples. The samples from South African were collected as part of a study on conservation genetics [19] and archived at the South African National Biodiversity Institute (SANBI) Wildlife Biobank. The study was approved by the SANBI Animal Research Ethics and Scientific Committee (ARESC), project number SANBI/RES/P18/29. Permission to do research under Section 20 of the Animal Diseases Act, 1984 (Act No. 35 of 1984) was granted by the Department of Agriculture, Land Reform and Rural Development (DALRRD), South Africa reference 12/11/1/1/1/18(1001) (JD). Furthermore, samples that were analyzed were collected from captive facilities under ethical approval from the University of the Free State, South Africa (Student project number: UFS-AED2016/0096) and the ARESC (SANBI/RES/P17/05) [19].

### Biological samples

A total of eight captive cranes from the Parc Zoologique de Paris and La Ménagerie du Jardin des Plantes Zoo in France were sampled and screened for haemosporidian parasites using microscopic examination and molecular characterization of the *cytb* gene. These included four Grey Crowned cranes (*B. regulorum*, ZB8232, ZB8231, ZB4382, and ZC1036), one East African Grey Crowned crane (*B. r. gibbericeps*, ZC1037), one West African Black Crowned crane (*B. p. pavonina*, ZC3162), one Blue crane (*A. paradiseus*, 10896), and one Demoiselle crane (*A. virgo*, MB7156). Two of the *B. regulorum* (ZB8232 and ZB8231) originated from a breeding program in France but were born to wild parents and imported as eggs or chicks into France before 2018. The other cranes were born and kept in captivity in zoological parks in France and/or other parts of Europe. The birds were sampled in February and July 2020 (ZB8232, ZB8231), in August 2023 (ZC1036, ZC1037, and ZC3162) and April and May 2024 (10896, MB7156) for veterinary purposes to assess and monitor their health status. Part of the blood samples was used to prepare two thin blood smears which were air-dried, and the remaining surplus blood was stored in EDTA tubes. All thin blood smears were fixed with absolute methanol prior to Giemsa staining (8% in phosphate-buffered solution) for 45 minutes. All EDTA blood tubes were frozen and stored at −20 °C until molecular analyses.

In South Africa, 147 whole blood samples were collected from captive-bred cranes in conservation facilities across three provinces, Gauteng Province, KwaZulu-Natal, and Eastern Cape from April 2017 to March 2018. The samples were from 134 adults and 13 juveniles and consisted of 76 females and 71 males. They represented 79 Blue cranes (*A. paradiseus*), 63 Grey Crowned cranes (*B. regulorum*), and 5 Wattled cranes (*B. carunculatus*). These samples were screened for haemosporidian parasites using molecular characterization of the *cytb* gene. No thin blood smears were available for these samples.

### Microscopic examination and parasite morphometry

The blood smears were carefully examined for parasite identification with a motorized BX63 Upright Olympus Microscope at a magnification of 1000× under oil immersion. An Olympus BX61 light microscope (Olympus, Tokyo, Japan) equipped with an Olympus DP70 digital camera and imaging software AnalySIS FIVE (Olympus Soft Imaging Solution GmbH, Münster, Germany) was used to examine preparations, prepare illustrations, and for parasite measurements. Haemosporidian parasites were morphologically identified according to Valkiūnas [61], and Valkiūnas and Iezhova [62]. Morphometric measurements were performed on 21 fully grown gametocytes.

### DNA extraction

Total genomic DNA of the French samples was extracted from 20 μL of whole blood samples using a QIAamp DNA Micro Kit (QIAGEN, Hilden, Germany), following the manufacturer’s instructions for the isolation of genomic DNA from small volumes of blood. Genomic DNA was extracted from approximately 40 μL of avian blood from the South African samples using a Quick-gDNA™ Mini Prep Extraction kit (Zymo Research, Inqaba Biotechnologies, Pretoria, South Africa). DNA concentration was determined using a NanoDrop 1000 spectrophotometer (Thermo Fisher Scientific, Waltham, MA, USA) after which the DNA was diluted to 25 ng/μL and stored at −20 °C pending PCR analysis.

### PCR and sequencing of the *cytb* barcode region

All French samples were screened for haemosporidian parasites using two nested PCR protocols targeting the mitochondrial *cytb* gene. The first PCR reactions with either H1 (5′–TGGTACTACAGGAGTAATGTTAGG–3′) and H2 (5′–CAATCGAGTTAACATGCTTAGACG–3′) primers or PLAS1 (5′–GAGAATTATGGAGTGGATGGTG–3′) and PLAS2a (5′–GTGGTAATTGACATCCWATCC–3′) were performed to amplify a 1,670 bp or 816 bp amplicon, respectively [21, 30]. PCR reactions were followed by nested PCRs using either H3 (5′–ATGTAATGCCTAGACGTATTCCTG–3′) and H4 (5′–GTTACCATAGCTGTTGATGGATG–3′) primers or PLAS3 (5′–GGTGTTTYAGATAYATGCAYGC–3′) and PLAS4 (5′–CATCCWATCCATARTAWAGCATAG–3′) to amplify a 1,369 bp (including the complete *cytb* gene) or 787 bp amplicon, respectively [21, 30]. The amplified fragments included the 478 bp barcode region of the *cytb* gene commonly used in haemosporidian parasite studies for lineage identification [8, 33].

All PCR and nested-PCR reactions with H1-H2-H3-H4 primers were performed in a final volume of 25 μL consisting of 5 μL of 5× PrimeSTAR GXL Buffer, 2 μL of dNTP Mixture (2.5 mM each), 0.5 μL of each primer (0.2 μM), 0.5 μL PrimeSTAR GXL DNA Polymerase (1.25 U) (Takara Bio, Shiga, Japan), 15 μL of nuclease-free water and 2 μL of DNA template. The PCRs included a denaturation at 98 °C for 10 s, followed by 40 cycles of amplification at 98 °C for 10 s, 60 °C for 15 s, 68 °C for 2 min (PCR reaction) or 45 s (nested-PCR reaction), and a final extension at 68 °C for 10 min. All PCR and nested-PCR reactions with PLAS1-PLAS2a-PLAS3-PLAS4 primers were performed in a final volume of 25 μL consisting of 2.5 μL of 10× reaction buffer B, 2.5 μL of dNTP Mixture (2 mM each), 2 μL of MgCl_2_ (25 mM), 2.5 μL of each primer (10 pmol/μL), 0.5 μL FIREPol^®^ DNA Polymerase (5 U/μL) (Solis Biodyne), 10.5 μL of nuclease-free water and 2 μL of DNA template. PCR and nested-PCR cycles were: denaturation at 94 °C for 5 min, followed by 40 cycles of amplification at 94 °C for 30 s, 55 °C for 30 s, 72 °C for 1 min, and a final extension at 72 °C for 10 min.

The nested PCR assay was used to amplify a fragment of 480 bp of the parasite *cytb* gene from the South African crane DNA samples [33]. The primary PCR was performed using 2 μL (~50 ng) template DNA, 12.5 μL Amplicon Red master mix (Lasec International, Cape Town, South Africa), 0.4 μM of primers HaemNF1 and HaemNR3 and molecular grade ddH2O to a final volume of 25 μL. The secondary PCR reaction contained 1 μL of the primary reaction, 0.4 μM primers HaemFL and HaemR2L (*Leucocytozoon* spp.) or primers HaemF and HaemR2 [8] (*Plasmodium* and/or *Haemoproteus* spp.), 12.5 μL Amplicon Red master mix and ddH_2_O to a final volume of 25 μL. Synthetic DNA gBlocks^®^ (Integrated DNA Technologies, Coralville, IA, USA; distributed by Whitehead Scientific (Pty) Ltd, Cape Town, South Africa) of *Plasmodium relictum* and *Leucocytozoon fringillinarum* (positive controls), and molecular grade water (negative control) were included in each PCR run. PCR cycling conditions were as follows: initial denaturation at 95 °C for 5 min, 20 cycles (primary PCR) and 35 cycles (nested PCRs) of denaturation at 94 °C for 30 s, annealing at 50 °C for 30 s, extension at 72 °C for 45 s, followed by a final extension at 72 °C for 10 min.

The PCR products were visualized on a 2% agarose gel. Amplicons were sequenced using one of the following methods: Sanger sequencing with PLAS3 and PLAS4 primers by Eurofins Genomics (France); Sanger sequencing with HaemFL and HaemR2L primers (targeting *Leucocytozoon* spp.) or HaemF and HaemR2 primers (targeting *Plasmodium* and/or *Haemoproteus* spp.) in-house at SANBI in South Africa, or NGS sequencing H3H4 amplicons.

Libraries were prepared according to the protocol described by Meyer and Kircher [41] and sequenced using a 500 cycles Kit v2 (2 × 250 bp, paired-end sequencing) on an Illumina MiSeq Illumina at the Service de Systématique Moléculaire (SSM), part of the Service Unit Données de Recherche pour l’Histoire Naturelle, l’Écologie & l’Environnement (UAR 2047 DoHNÉE) at the Muséum National d’Histoire Naturelle in Paris. All sequences obtained with Sanger sequencing were viewed, edited, and checked for ambiguous nucleotides in the sequences with CHROMAS software (Technelysium Pty Ltd, South Brisbane, QLD, Australia) or CLC Main Workbench programme (CLC Bio, Boston, MA, USA). Consensus sequences were generated and aligned with published reference sequences using MEGA11: Molecular Evolutionary Genetics Analysis version 11 (Tamura, Stecher, and Kumar 2021). FastqR1 and fastqR2 paired reads obtained from Illumina sequencing from each sample were aligned against complete *cytb* reference sequences retrieved from GenBank (NCBI) and MalAvi database (http://130.235.244.92/Malavi/) using Burrows-Wheeler Aligner (BWA) software [40]. The alignments were visualized using Integrative Genomics Viewer (IGV) (Cambridge, MA, USA), v2.13.2 [53]. Reliable consensus *cytb* sequences from *Haemoproteus* parasite lineages were then manually generated.

New *cytb* sequences (hBALREG01, hBALREG02, hANTPAR02, hBAREGI03, hBAREGI04, hBAREGI05, hBAREGI07, pANTPAR03, pANTPAR04, pANTPAR05, pBAREGI09, lBAREGI01, and lBAREGI02) were deposited in GenBank (accession numbers PV708087 and PV708088 for hBALREG01 and hBALREG02, respectively, and OL330700–OL330736 for the others) and MalAvi databases.

### DNA haplotype network

A DNA haplotype network was constructed using the 475 bp *cytb* lineage sequences of the new *Haemoproteus* lineages to visualize their relatedness, distribution among different bird hosts, and geographical substructure patterns. The analysis included all new *Haemoproteus cytb* sequences from Gruidae birds in South Africa and France. Unique *Haemoproteus* lineages (*cytb* haplotypes) previously identified in Gruidae birds from other studies were retrieved from GenBank and MalAvi databases and included in the analysis. A Median Joining haplotype network was generated with PopART v1.7 using default settings [2]. The networks were graphically prepared and annotated with host species and country information using Inkscape v1.0.1.

### Phylogenetic analysis

To assess the phylogenetic position and relationships of *Haemoproteus* parasites belonging to the *Haemoproteus* group of Gruidae birds within the three genera of haemosporidian parasites, a phylogenetic tree was constructed based on a final alignment containing a total of 157 available *cytb* sequences of haemosporidian parasites (475 bp) downloaded from GenBank, including the *cytb* lineage of *H. balearicae* and other *cytb* lineages of *Haemoproteus* parasites from Gruidae birds. From this dataset, sequences of *Haemoproteus (Haemoproteus)* species, as well as those providing information on vertebrate hosts, were primarily selected to provide a robust framework for understanding host-parasite specificity and evolutionary relationships. The sequences were aligned using the ClustalW program on MEGA 11 software [59]. The substitution models for the dataset were evaluated using IQ-TREE v1.6.12 [43]. Based on the corrected Akaike Information Criterion (cAIC), the best substitution model for all alignments was determined to be GTR + I + G. A Maximum Likelihood (ML) tree was constructed in IQ-TREE v1.6.12 [43], with 10,000 bootstrap replicates. Bayesian analyses were conducted using MrBayes v3.2.7a [54] over a total of ten million generations, with sampling every 100 generations. The first 25% of the trees were discarded as burn-in before constructing the consensus tree. The resulting phylogenetic trees were visualized using FigTree v1.4.4 and further annotated and displayed using Inkscape, v1.0.1 (Inkscape Project). Trees were rooted with avian *Leucocytozoon* parasites.

## Results

### Morphological characteristics of *Haemoproteus* parasites

A total of three blood samples from captive grey crowned cranes (ZB8232, ZB8231, and ZC1037) housed in France were positive for *Haemoproteus* parasites after microscopic examination. After a deep microscopic examination of all positive blood films, the *Haemoproteus* parasite morphospecies was identified as *Haemoproteus balearicae* [50] based on its morphological characteristics.

**Figure 1:**
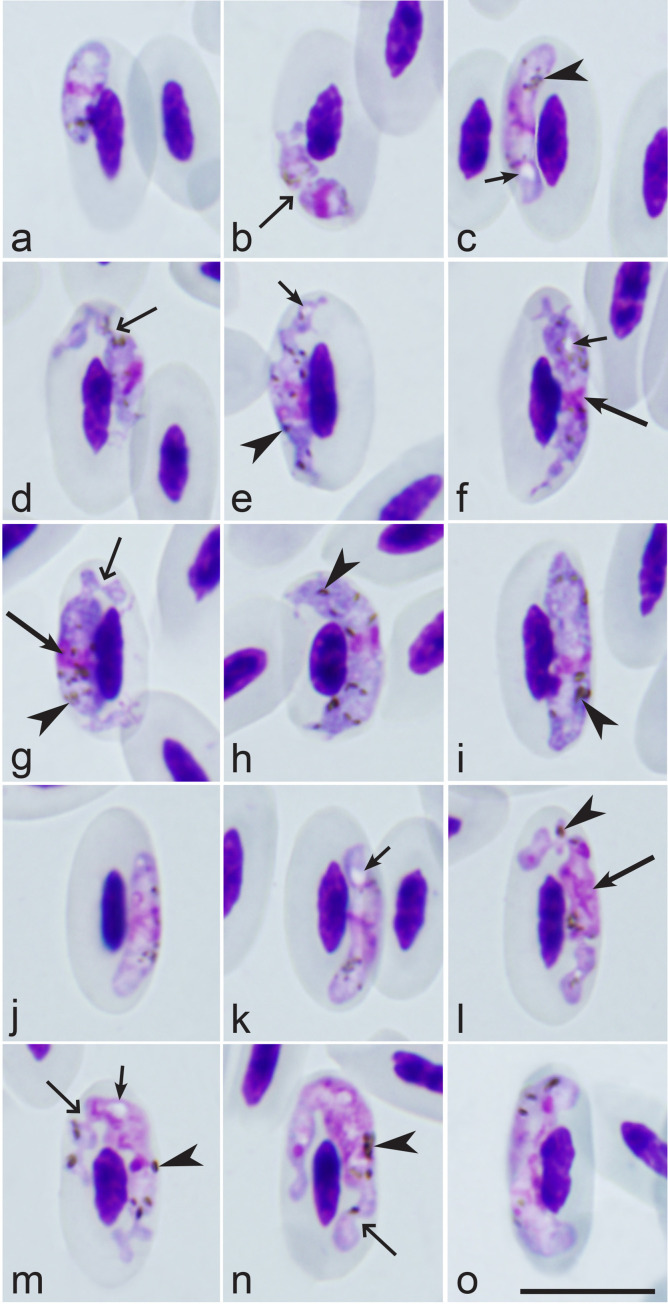
Gametocytes of *Haemoproteus balearicae* (lineage hBAREGI210) from the blood of grey crowned crane *Balearica regulorum*: (a)–(i) – macrogametocytes, (j)–(o) – microgametocytes. Note: the slender form and markedly irregular gametocyte outlines, with numerous deep indentations providing various lobular-like appearances to the parasites (d, g, l–n). Long simple arrows – parasite nuclei; simple arrowheads - pigment granules; simple wide long arrows – indentations; short simple arrows – vacuoles. Giemsa-stained thin blood films. Scale bar = 10 μm.

Morphological characterization of *H. balearicae* Peirce, 1973, the parasite lineage hBAREGI210 found in an additional (non-type) host, *Balearica regulorum* (Figs. 1a–1o, Table 1).

**Table 1:** Morphometry of host cells and mature gametocytes of *Haemoproteus balearicae* (lineage hBAREGI210) from the blood of *Balearica regulorum*.

Feature	Measurements (μm) ^a^
Uninfected erythrocyte	
Length	13.3–16.0 (15.0 ± 0.7)
Width	5.7–7.5 (6.8 ± 0.5)
Area	65.7–96.7 (82.7 ± 8.1)
Uninfected erythrocyte nucleus	
Length	4.8–7.3 (6.2 ± 0.7)
Width	1.7–2.7 (2.2 ± 0.3)
Area	8.9–14.8 (11.8 ± 1.7)
Macrogametocyte	
Infected erythrocyte	
Length	13.9–16.9 (15.3 ± 0.9)
Width	6.0–8.3 (7.4 ± 0.6)
Area	76.2–112.5 (91.1 ± 9.0)
Infected erythrocyte nucleus	
Length	5.4–7.4 (6.1 ± 0.5)
Width	1.8–2.9 (2.3 ± 0.3)
Area	10.1–14.2 (11.4 ± 0.9)
Gametocyte	
Length	12.8–18.9 (15.6 ± 1.7)
Width	1.8–3.4 (2.7 ± 0.4)
Area	25.6–38.1 (31.9 ± 3.8)
Gametocyte nucleus	
Length	2.0–3.6 (2.6 ± 0.5)
Width	0.9–2.7 (1.7 ± 0.4)
Area	2.4–4.9 (3.6 ± 0.7)
Pigment granules	9.0–16.0 (12.0 ± 2.3)
NDR ^b^	0.6–1.0 (0.9 ± 0.1)
Microgametocyte	
Infected erythrocyte	
Length	13.6–17.7 (15.8 ± 1.2)
Width	6.6–9.0 (7.3 ± 0.6)
Area	80.2–104.5 (92.6 ± 8.4)
Infected erythrocyte nucleus	
Length	5.4–8.4 (6.6 ± 0.8)
Width	1.8–2.7 (2.1 ± 0.3)
Area	9.7–13.9 (11.6 ± 1.1)
Gametocyte	
Length	15.8–23.9 (20.7 ± 2.3)
Width	2.3–3.7 (2.9 ± 0.4)
Area	28.2–48.3 (39.8 ± 5.3)
Gametocyte nucleus	
Length	–
Width	–
Area	–
Pigment granules	7.0–9.0 (8.0 ± 0.7)
NDR	0.8–1.0 (0.9 ± 0.1)

a All measurements (*n* = 21) are given in micrometers. Minimum and maximum values are provided, followed in parentheses by the arithmetic mean and standard deviation.

b NDR = nucleus displacement ratio according to Bennett and Campbell (1972).

The main diagnostic characteristics of *H. balearicae* from *B. regulorum* align with former descriptions [50, 61]. This study reports the first complete *cytb* sequences for this parasite (hBALREG01 and hBALREG02) and, for the first time, associates the previously published *cytb* lineage hBAREGI210 with a specific species. The main features of blood stages of the hBAREGI210, hBALREG01, and hBALREG02 lineages are as follows.

Young gametocytes (Figs. 1a–1d, 1j, 1k): The earliest forms can be seen anywhere in infected erythrocytes. Growing gametocytes predominantly take subpolar position in the host cells (Figs. 1a, 1b, 1d), but advanced gametocytes were also often seen in lateral position to the nuclei (Fig. 1c). U-shaped gametocytes, which assume various polar or sub-polar positions in erythrocytes are common (Fig. 1d). Growing gametocytes are often slender, they do not touch or slightly touch the nuclei or envelope of erythrocytes or both these host cell structures (Figs. 1b, 1c). Single small vacuoles were seen occasionally in advanced gametocytes. The parasite nucleus is small, often band-like (Figs. 1a–1c). Gametocyte outlines vary from even (Figs. 1a, 1c) to markedly ameboid or lobulated due to prominent indentations, which provide a lobular-like appearance to such cells (Figs. 1b, 1d). Pigment granules are small, predominantly roundish and are grouped together (Fig. 1c). Influence on host cells is not pronounced.

Macrogametocytes (Table 1; Figs. 1e–1i): The cytoplasm is heterogeneous in appearance and sometimes contains a few small vacuoles (Figs. 1e, 1f). Gametocytes assume a lateral position to the nuclei of infected erythrocytes and only negligibly (if at all) enclose them with their ends (Figs. 1e–1i). Fully grown gametocytes adhere to both the nuclei and the envelope of erythrocytes. Gametocyte outline is predominantly markedly ameboid (Figs. 1e, 1f, 1h) or wavy-lobulated with prominent indentations on their ends (Fig. 1g). Fully grown gametocytes gradually lose their ameboid outline and become even (Figs. 1i). The parasite nucleus is compact, small (Table 1; Figs. 1f–1i), variable in form, predominantly median or submedian in position. Pigment granules are mostly randomly scattered, sometimes grouped (Figs. 1g, 1i), of medium size (0.5–1.0 μm); they were predominantly roundish in growing gametocytes (Figs. 1e–1g), but often assumed oval or slightly elongate in fully grown gametocytes (Figs. 1h, 1i). Gametocyte influence on host cell nuclei is not pronounced.

Microgametocytes (Table 1; Figs. 1j–1o): The general configuration and other features are as for macrogametocytes with the usual sexual dimorphic characters, which are paler staining of the cytoplasm and large diffuse nuclei. Vacuoles (Figs. 1k, 1m) are more numerous and bigger than in the macrogametocytes.

A voucher blood preparation of *H. balearicae* lineage, hBAREGI210 (accession no. MNHN-IR-2025-03, date, Parc Zoologique de Paris) was deposited at the Muséum National d’Histoire Naturelle, Paris. Representative DNA sequences: Mitochondrial *cytb* complete sequences hBALREG01 (including hBAREGI210 barcode) and hBALREG02 (GenBank accession numbers PV708087–PV708088).

### Molecular analyses

DNA samples from all eight French cranes were analyzed using PCR, next-generation sequencing, and Sanger sequencing to detect haemosporidian parasites and to identify and characterize *H. balearicae* at the molecular level. Among these, three samples (ZB8232, ZB8231, and ZC1037), in which *H. balearicae* had previously been observed in blood smears, tested positive by molecular methods (37.5% prevalence) and were successfully sequenced. Microscopic examination had the same sensitivity in the detection of the parasites as molecular identification.

Nested PCR analysis of 147 cranes, from two South African localities, Eastern Cape and Gauteng Province, revealed that 55 cranes (molecular prevalence of 37.4%) were positive for at least one haemosporidian lineage (Supplementary Table 1). No infections were identified from the two cranes from KwaZulu-Natal. The overall prevalence of haemosporidian parasites was 47.5% in the Eastern Cape and 34.3% in Gauteng Province. Prevalence varied between the three crane species, with rates of 22.2% in Wattled cranes, 27.8% in Blue cranes, and 52.5% in Grey Crowned cranes (Supplementary Table 1). Most prevalently infected cranes were adults (53/55), while only two juveniles (one male and one female) from Gauteng Province tested positive for haemosporidian parasites, identified as either *Plasmodium* or *Haemoproteus* spp. There was no significant difference in prevalence between sexes, as males and females were equally affected. The molecular prevalence of *Plasmodium*/*Haemoproteus* spp. (41/55, 74.5%) was higher than that of *Leucocytozoon* spp. (7/55, 12.7%), regardless of location or crane species (Supplementary Table 2). Of the 55 PCR-positive samples, 37 were successfully sequenced by Sanger sequencing, identifying 22 *Haemoproteus* spp., nine *Plasmodium* spp. and six *Leucocytozoon* spp. Among these, there were six co-infections with *Leucocytozoon* spp. and *Haemoproteus* spp., and one co-infection with *Leucocytozoon* spp. and an undetermined *Haemoproteus*/*Plasmodium* spp.

Two very similar complete *cytb* sequences hBALREG01 and hBALREG02 (1379 bp), were identified from the three French crane samples. These sequences differed by only two single nucleotide polymorphisms (SNPs) across the complete *cytb* sequence and by a single SNP when comparing the 478 bp *cytb* region. hBALREG01 was detected in all three samples, whereas hBALREG02 was only found in sample ZB8231, co-occurring with the hBALREG01 sequence. In Shen *et al.* [57], the published *cytb* sequence (OR662132) contained a nucleotide code “Y”, representing either a cytosine (C) or thymine a (T), which corresponds to the polymorphism observed between hBALREG01 and hBALREG02. Additionally, hBALREG01 was identical to the published *cytb* lineage hBAREGI210. A total of 37 amplicons were successfully sequenced from South African Gruidae, yielding 15 distinct *cytb* sequences (Table 2). The hBAREGI210 lineage, detected in *B. regulorum* and *B. r. gibbericeps* from French cranes and in *A. paradiseus* and *B. regulorum* from South African cranes, was recorded and published in *B. r. gibbericeps* from Rwanda in Sobeck *et al.* [58] and assigned to *Haemoproteus antigonis*. However, the morphology of this parasite lineage was not thoroughly investigated in this study. In the present study, hBAREGI210, together with hBALREG01 and hBALREG02, was confidently linked to *Haemoproteus balearicae*. The hBAREGI210 lineage showed a close similarity to other *cytb* lineages, differing by 2 SNPs (0.4%) from the hBAREGI07 lineage and by 4 SNPs (0.8%) from the hBAREGI03, hBAREGI04, and hBAREGI05 lineages, all identified in South African *B. regulorum* samples (Table 2). The *H. balearicae* hBAREGI210 *cytb* lineage was molecularly divergent from other *Haemoproteus cytb* sequences from Gruidae species, with differences ranging from 25 SNPs (5.2%) to 40 SNPs (8.4%). Some of these lineages have been previously published and linked to *H. antigonis* [10, 37, 58, 67]. The minimum average genetic distance between the 478 bp *cytb* lineage sequences of lineages within the *H. antigonis* species group and other haemosporidian parasites (including other *Haemoproteus* species) was approximately 8%. Two additional *Haemoproteus cytb* lineages were identified in South African Gruidae. The hANTPAR02 lineage was detected in an *A. paradiseus*, while the hBAREGI046 lineage was found in both *A. paradiseus* and *B. regulorum* birds. Previously reported from *B. r. gibbericeps* in Rwanda [58], hBAREGI046 was assigned to *H. antigonis*. The newly identified hANTPAR02 lineage differed from hBAREGI046 by 4.2% (20/475 bp) and from hBAREGI05 by 6.7% (32/474 bp). Also, *B. regulorum* showed the highest *Haemoproteus* prevalence and *cytb* lineages diversity, with six distinct *cytb* lineages, compared to *A. paradiseus* and *B. carunculatus* (Table 2).

**Table 2:** *Cytb* lineages of haemosporidian parasites identified from South African and French cranes.

*Cytb* barcodes/MalAvi Lineage	Host	Locality	BLAST (GenBank or MalAvi)	
hBAREGI210, **hBALREG02**	Blue crane (*Anthropoides paradiseus*), Grey-crowned crane (*Balearica regulorum*); East African Grey Crowned crane (*Balearica regulorum gibbericeps*)	GP, EC	*Haemoproteus balearicae* (BAREGI210)	100%
**hBAREGI03**	Grey-crowned crane (*Balearica regulorum*)	GP	*Haemoproteus balearicae* (BAREGI210)	99,34%
**hBAREGI04**	Grey-crowned crane (*Balearica regulorum*)	GP	*Haemoproteus balearicae* (BAREGI210)	99,34%
**hBAREGI05**	Grey-crowned crane (*Balearica regulorum*)	GP	*Haemoproteus balearicae* (BAREGI210)	99,34%
**hBAREGI07**	Grey-crowned crane (*Balearica regulorum*)	GP	*Haemoproteus balearicae* (BAREGI210)	99,78%
**hANTPAR02**	Blue crane (*Anthropoides paradiseus*)	GP	*Haemoproteus antigonis* (BAREGI046)	96,00%
hBAREGI046	Blue crane (*Anthropoides paradiseus*), Grey-crowned crane (*Balearica regulorum*)	GP, EC	*Haemoproteus antigonis* (BAREGI046)	100%
pCATUST05	Blue crane (*Anthropoides paradiseus*)	GP	*Plasmodium lutzi* (CATUST05)	100%
**pANTPAR03**	Blue crane (*Anthropoides paradiseus*)	GP	*Plasmodium* sp. (CXPER01)	99,00%
**pANTPAR04**	Blue crane (*Anthropoides paradiseus*)	GP	*Plasmodium* sp. (CXPER01)	99,14%
**pANTPAR05**	Blue crane (*Anthropoides paradiseus*), Wattled crane (*Bugeranus carunculatus*)	GP	*Plasmodium* sp. (CXPER01)	99,37%
**pBAREGI09**	Grey-crowned crane (*Balearica regulorum*), Wattled crane (*Bugeranus carunculatus*)	GP	*Plasmodium relictum* pGRW04	99,15%
lCIAE02	Blue crane (*Anthropoides paradiseus*)	GP, EC	*Leucocytozoon* sp. CIAE02	100%
lBAREGI252	Grey-crowned crane (*Balearica regulorum*)	GP, EC	*Leucocytozoon* sp. BAREGI252	100%
**lBAREGI02**	Grey-crowned crane (*Balearica regulorum*)	EC	*Leucocytozoon* sp. BAREGI252	99,79%

*Cytb* lineages in bold are newly detected; others were previously reported.

BC – Blue crane, *Anthropoides paradiseus*; GC – Grey crowned crane, *Balearica regulorum*; WC – Wattled crane *Bugeranus carunculatus*; GP – Gauteng Province; EC – Eastern Cape.

Five *Plasmodium cytb* lineages were identified in Gruidae birds in South Africa (Table 2), all originating exclusively from Gauteng Province. The pCATUST05 lineage, identified in *A. paradiseus*, belongs to the *Plasmodium lutzi* group, as previously defined in Harl *et al.* [28].

Four *cytb* lineages (pANTPAR03, pANTPAR04, pANTPAR05, and pBAREGI09) were newly detected from Gruidae birds in this study. The pBAREG09 lineage showed close genetic similarity to pLINOLI03, pFOUMAD03, and *P. relictum* pGRW04. The lineages pANTPAR03–pANTPAR05 were closely related, differing by only 1–3 SNPs, and shared 3–5 SNPs differences with *Plasmodium* sp. pCXPER01 (HM179147), identified in *Culex* spp. This parasite has not been assigned to a specific morphospecies.

Three *Leucocytozoon cytb* lineages (lCIAE02, lBAREGI252, and lBAREGI02) were identified in Gruidae birds from the Eastern Cape and Gauteng Provinces of South Africa (Table 2). The lCIAE02 lineage, found in *A. paradiseus*, was previously published and referred to as *Leucocytozoon “aff.” californicus* (species “affinis”). This lineage showed close genetic similarity to the lFASPAR02 lineage, differing by only two SNPs over a 475 bp sequence. The lFASPAR02 lineage had already been linked to the morphospecies *L. californicus* [65]. The lBAREGI252 and lBAREGI02 lineages, both identified in *B. regulorum*, were closely related, differing by only one SNP. They differed from the lCIAE02 lineage by 31 (6.5%) and 30 (6.3%) nucleotides, respectively. The lBAREGI252 lineage had previously been reported in *B. r. gibbericeps* from Rwanda but has not yet been linked to a specific morphospecies [58].

### Haplotype network of *Haemoproteus* group *cytb* sequences of Gruidae

**Figure 2:**
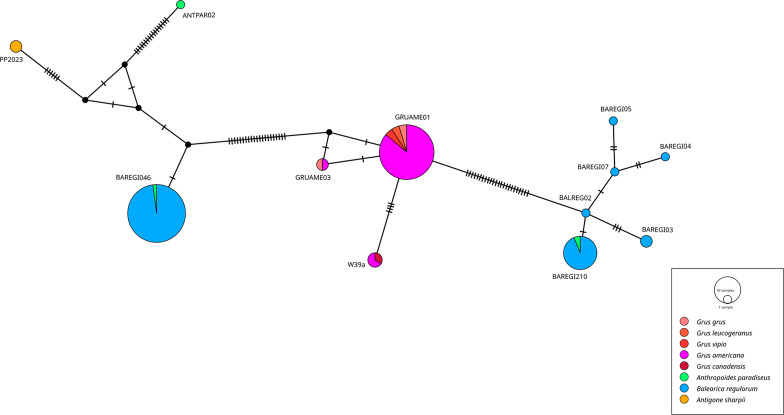
Median-Joining DNA haplotype network showing the host and geographic distribution of twelve *Haemoproteus antigonis* and *H. balearicae* lineages (475 bp c*ytb* sequences) found in Gruidae birds.

To further explore the genetic structure and diversity of *Haemoproteus* lineages infecting Gruidae, a DNA haplotype network was constructed based on *Haemoproteus cytb* sequences (475 bp) identified in the Gruidae birds, including cranes in South Africa and France (Fig. 2). The *Haemoproteus* lineages clustered into three distinct subclades, differing by up to 67 bp from each other. The genetic distance among the three groups ranged from 10.1% to 14.1%. The first group included *H. antigonis* hPP2023, hANTPAR02, and hBAREGI046 lineages, detected in different crane genera, *A. paradiseus*, *A. sharpii*, and *B. regulorum* across South Africa, Rwanda, and Thailand. The second group comprised the *H. antigonis* hGRUAME01, hGRUAME03, and hW39a, all found in five species of the *Grus* genus (*G. grus*, *G. leucogeranus*, *G. vipio*, *G. americana*, and *G. canadensis*) from North America (Canada and USA) and Asia (China). The third group consisted of *H. balearicae* (hBAREGI210, hBALREG02, hBALREGI03, hBALREGI04, hBALREGI05, and hBALREGI07), primarily found in *B. regulorum*, except for one in *A. paradiseus*. These lineages were identified in cranes from South Africa, Rwanda, France, and China (imported cranes). This haplotype network illustrates both the genetic distinctiveness of major *Haemoproteus* groups infecting cranes and their distribution across different hosts and regions.

### Phylogenetic analyses

**Figure 3:**
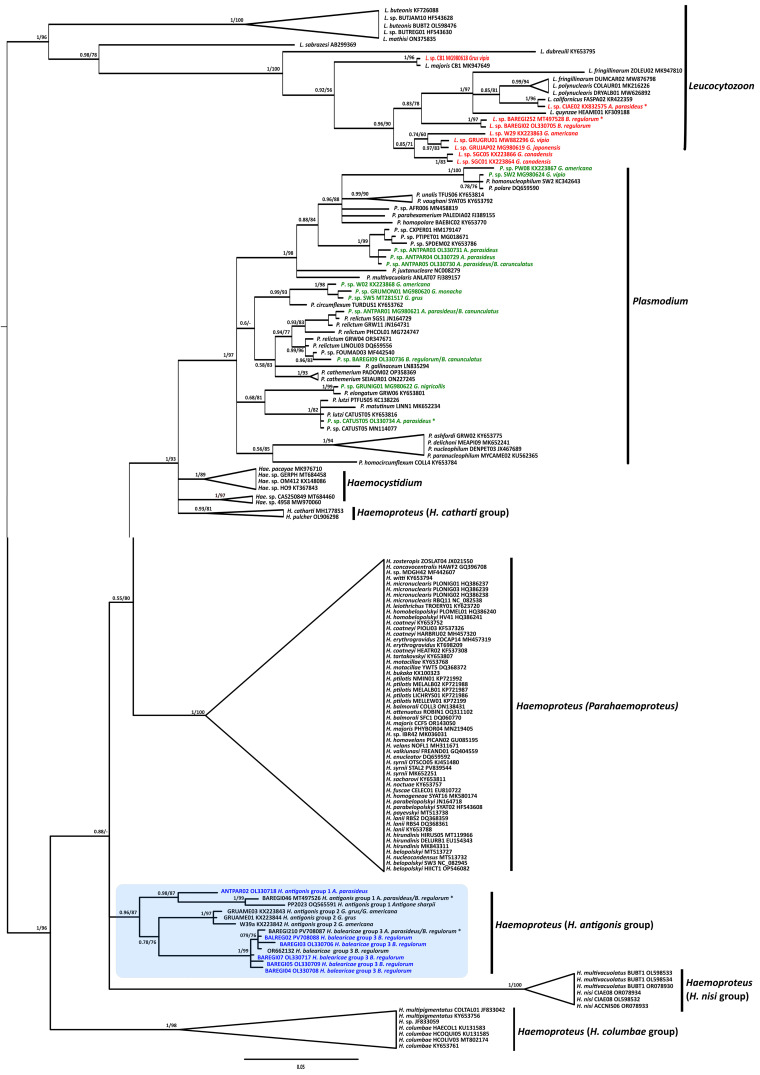
Phylogenetic tree based on 475 bp of *cytb* sequences from 157 haemosporidian lineages reconstructed using both Maximum Likelihood (ML) and Bayesian Inference (BI) methods. The trees were rooted using *Leucocytozoon* parasites. Node support values are indicated as Bayesian posterior probability/ML bootstrap support. Sequences of *Leucocytozoon* from Gruidae birds are highlighted in red, with bold red indicating parasite lineages newly identified in this study, and regular red indicating parasite lineages detected in previous studies (an asterisk (*) marks those also found in this study). Plasmodium sequences from Gruidae birds are shown in green, with bold green indicating parasite lineages newly identified in this study, and regular green indicating parasite lineages detected in previous studies (an asterisk (*) marks those also found in this study). Sequences from the *Haemoproteus antigonis* group are enclosed in a blue frame and include both previously described lineages (an asterisk (*) marks those also found in this study) and newly identified lineages from cranes, which are shown in bold blue.

Both Bayesian Inference (BI) and Maximum Likelihood (ML) analyses produced similar topologies, and support values from both methods are indicated at the corresponding nodes in the phylogenetic tree (Fig. 3). The *Haemoproteus cytb* lineages identified in cranes formed a distinct and well-supported clade within the broader haemosporidian parasite phylogeny (BI posterior probability: 0.96/ML bootstrap support: 87) as shown in Figure 3. Within this clade, three well-supported phylogenetic subgroups were identified. Group 1 consisted of *Haemoproteus cytb* sequences linked to *H. antigonis* from the genera, *Anthropoides*, *Antigone*, and *Balearica*, from Africa (South Africa and Rwanda) and Asia (Thailand) (BI: 0.98/ML bootstrap support: 87). Group 2 included *Haemoproteus cytb* sequences, also linked to *H. antigonis*, from *Grus* species from North America (Canada and USA) and Asia (China) (BI posterior probability: 1/ML bootstrap support: 97). Group 3 consisted of *H. balearicae* lineages and closely related lineages, found primarily in *B. regulorum* and *A. paradiseus* from South Africa, Rwanda, France, and China (imported cranes) (BI posterior probability: 1/ML bootstrap support: 99). The *Plasmodium* lineage pCATUST05, identified in *A. paradiseus* from South Africa grouped with the *P. lutzi* lineage (KY653816) found in the Black-and-white Warbler (*Mniotilta varia*) and the *P.* sp. lineage (MN114077) detected in the Golden-winged Warbler (*Vermivora chrysoptera*), both passerine birds from North America. It also grouped with the *P. lutzi* pPTFUS05 lineage (KC138226) found in the passerine Great Thrush (*Turdus fuscater*) from South America (BI: 1/ML: 82).

The pBAREGI09 lineage, identified in *B. regulorum* and *B. carunculatus*, grouped within a clade composed of four lineages of *P. relictum* (pSGS1, pGRW11, pLZFUS01, and pPHCOL01) (BI: 0.94/ML: 77), and more specifically with lineages pLINOLI03, pFOUMAD03, and *P. relictum* pGRW04. The pANTPAR03 and pANTPAR04 lineages, found in *A. paradiseus*, and the pANTPAR05 lineage, found in both *A. paradiseus* and *B. carunculatus*, grouped together and with *Plasmodium* pCXPER01 (HM179147), pPTIPET01 (MG018671), and pSPDEM02 (KY653786) lineages (BI: 1/ML: 99). These lineages have not yet been assigned to a morphospecies. This group was placed within a broader clade with *Plasmodium* lineages previously identified in cranes in North American and Asia.

Regarding *Leucocytozoon*, the *L. aff. californicus* lCIAE02 lineage grouped with a well-supported phylogenetic group with *L. californicus* lFALSPAR02 lineage and other lineages related to *L. “fringillinarum”*, *L. polynuclearis*, and *L. quynzae* (BI posterior probability: 1 /ML bootstrap support: 97). The lBAREGI252 and lBAREGI02 lineages grouped together and formed a distinct group. These lineages were part of a broader clade and included five *Leucocytozoon* lineages previously identified in cranes from North American and Asia (BI posterior probability: 0.96/ML bootstrap support: 90).

## Discussion

The primary objectives of this study were to identify and characterize haemosporidian parasites of Gruidae birds, to generate new sequence data for these parasites in cranes, and to characterize the morphology of the blood stages of *Haemoproteus*. Molecular analyses and blood smears revealed infections with *Haemoproteus*, *Plasmodium*, and *Leucocytozoon* parasites, highlighting variations in prevalence and parasite diversity among cranes. Four Haemosporida species have previously been recorded in Gruidae birds: *H. antigonis*, *H. balearicae*, *P. polare*–like, and *L. grusi* [5, 6, 20]. Among these, only *H. antigonis* has been partially characterized morphologically and associated with *cytb* sequences [10]. However, a complete morphological redescription including all developmental stages is still lacking. Furthermore, several *Haemoproteus*, *Plasmodium*, and *Leucocytozoon cytb* lineages have been reported from Gruidae birds in the USA, China, and Rwanda [9, 10, 37, 57, 58]. All the *Haemoproteus cytb* lineages reported so far have been assigned to *H. antigonis*, while the *Plasmodium* and *Leucocytozoon cytb* lineages remain morphologically unidentified.

*Haemoproteus balearicae* was originally described in wild *B. pavonina* imported from West Africa [50], but a combined molecular and morphological characterization has not been conducted until now. In this study, *H. balearicae* was re-described morphologically in *B. regulorum* and characterized molecularly, and was linked to the *cytb* lineages hBAREGI210, hBALREG01, and hBALREG02. Importantly, *H. balearicae* hBAREGI210 can be readily distinguished from other haemoproteids parasitizing Gruidae birds, particularly by its fully grown gametocytes, which are elongated and slender, ameboid in outline (Figs. 1e, 1f, 1m). The *H. balearicae* hBAREGI210 lineage was predominantly found in captive *B. regulorum* in France and South Africa*,* suggesting that *B. regulorum* is a common host for this species. This *cytb* lineage was also identified in one *A. paradiseus* in South Africa, indicating that the host range of *H. balearicae* may be broader. In addition, this study reports the presence of *H. balearicae* in both captive South African cranes and captive-born cranes in France, suggesting a broader geographic distribution than previously recognized and highlighting its presence in Europe. The detection of *H. balearicae* in captive-born cranes in France further implies that local transmission is occurring, likely involving indigenous dipteran vectors. This raises important questions regarding potential local transmission cycles in captive settings in France. The hBAREGI210 lineage was initially identified as *H. antigonis* by Sobeck *et al.* [58]. The authors reported no significant morphological differences between *Haemoproteus* parasites observed in their study and the morphological characteristics of *H. antigonis* described in previous studies [9, 10, 61]. However, the authors raised the possibility of cryptic speciation within the *H. antigonis* morphospecies, as they found another *Haemoproteus cytb* lineage, hBAREGI046, also referred to as *H. antigonis* in their study that differed from hBAREGI210 by 32 bp. In the present study, hBAREGI210 has been confidently reassigned to the *H. balearicae* morphospecies. Furthermore, this study reported four additional *Haemoproteus cytb* lineages (hBAREGI03, hBAREGI04, hBAREGI05, and hBAREGI07), which were molecularly closely related to *H. balearicae* hBALREGI210 and mainly found in *B. regulorum* in South Africa. These findings suggest the possibility of greater molecular diversity within *H. balearicae* or the presence of closely related *Haemoproteus* parasites infecting cranes. The results of the current and previous studies indicate that the molecular diversity of *Haemoproteus* parasites in cranes is greater than previously recognized.

Molecular and phylogenetic analysis revealed at least three distinct groups of *Haemoproteus* parasites in cranes (Gruidae), suggesting that they may represent at least three distinct parasite species. The first group consists of *H. balearicae* hBAREGI210 and closely related *cytb* lineages predominantly found in *B. regulorum* in Africa (South Africa and Rwanda), France and China (imported bird). The second group includes the hBAREGI046 and hANTPAR02, mainly found in *B. regulorum* in Africa, as well as the hBP013 lineage from *A. antigone* in Thailand. The third group encompasses three lineages identified in *Grus* species in North America and China. The parasites in the latter two groups have been identified as *H. antigonis* [9, 10, 37]. These groups formed two well-supported phylogenetic clusters and were molecularly distant from one another, suggesting that they likely represent two distinct *Haemoproteus* species. A redescription and molecular characterization of *H. antigonis* is needed, ideally from its vertebrate type host, the Demoiselle crane (*A. virgo*), sampled near the type locality in Junagadh, India, to accurately determine its group affiliation.

All *Haemoproteus* parasites identified in Gruidae birds formed a distinct and monophyletic phylogenetic clade within the haemosporidian parasite phylogeny, separate from other described *Haemoproteus* clades. Notably, the presence of *H. balearicae* and *H. antigonis* has been reported exclusively in Gruidae birds, underscoring their potential host specificity within birds of this family. Parasites within the *H. balearicae* and *H. antigonis* clade may warrant reclassification as a distinct subgenus or even a separate genus. However, their current taxonomic status remains unchanged until further information is available regarding their exo-erythrocytic stage development in avian hosts, as well as their transmission and life cycle in dipteran vectors, which remain unknown. This study highlights the critical need for basic parasitological research to deepen our understanding of haemosporidian parasite diversity, taxonomic status, and host-vector relationships.

The findings of this study also reveal the presence of five distinct *Plasmodium* and three *Leucocytozoon cytb* lineages in South African Gruidae. The pCATUS05 belonging to *P. lutzi* group, was identified in *A. paradiseus* from Gauteng Province. *Plasmodium lutzi* Lucena, 1939 was first described in Grey-cowled Wood Rail (*Aramides cajaneus*; Rallidae, Gruiformes) in Brazil. According to MalAvi records, the *P. lutzi* pCATUS05 lineage has been identified in various Passeriformes species (Turdidae, Paridae, Passerellidae, Troglodytidae, Parulidae, Thraupidae) and non-Passeriformes species (Strigidae), exclusively from South and North America and the Caribbean region [28]. This is the first record of the pCATUS05 lineage from Gruidae birds from South African localities, highlighting that the geographic and host range of the *P. lutzi* lineage is broader than previously recognized. *Plasmodium* lineage (pBAREGI09) identified in *B. regulorum* and *B. carunculatus* was genetically closely related to *P. relictum* lineages pGRW04, *P*. spp. pFOUMAD03, and pLINOLI03. The *P. relictum* pGRW4 and pSGS1 lineages have previously been reported from cranes [37, 64]. Both lineages have a wild host and a geographical distribution and have been reported to be actively transmitted mainly in America and southern Africa. The pANTPAR01 lineage, previously reported in *A. paradiseus* and *B. carunculatus* (Gruidae) by Jia *et al.* [37], was also classified within the *P. relictum* group, potentially expanding the molecular diversity of *P. relictum* sequences. However, to confidently associate pBAREGI09 and pANTPAR01 as new lineages of the *P. relictum* species, further comparisons of the morphological characteristics of blood stages would be necessary. *Plasmodium relictum* commonly infects zoo birds around the world. Penguins are highly susceptible to infection by this parasite and infection usually results in severe disease and mortality [7]. In South Africa, *P. relictum* infections have been reported from wild birds and mosquitoes on the southern coast of the country (Eastern and Western Cape Provinces) [46, 47, 55].

The *Plasmodium* lineages pANTPAR03, pANTPAR04, and pANTPAR05, were closely related and grouped phylogenetically with the pCXPER01 *Plasmodium* lineage found in the Tawny pipit, a small migratory passerine of the family Motacillidae, Passeriformes [11], and *Culex* spp. [24, 45]. They were also closely related with pPTIPET01 and pSPDEM02 *Plasmodium* lineages found in other bird families, namely Phasianidae (Stone partridge, *Ptilopachus petrosus*) in Benin and Spheniscidae (African penguin, *Spheniscus demersus*) in South Africa [31, 48]. However, there was no morphological description available from the South African Gruidae sample in this study, and all these *cytb* lineages are not yet linked to morphospecies.

Three distinct *Leucocytozoon cytb* lineages (lCIAE02, lBAREGI252, and lBAREGI02) were identified in South African Gruidae in this study. The lCIAE02 lineage referred to *Leucocytozoon “aff.” californicus* was first described in the crane *A. paradiseus*. Previously, this lineage was reported as a common parasite of raptors (including Accipitriformes, Falconiformes, and Strigiformes) and was found infecting birds from seven other orders across diverse regions of the world, including Europe, Asia, and Africa [16, 29, 35, 38, 51, 67]. In South Africa, the lCIAE02 lineage was previously identified in the Woodland Kingfisher (*Halcyon senegalensis*) and the Diederik Cuckoo (*Chrysococcyx caprius*) in Limpopo Province by Chaisi [15]. However, this *Leucocytozoon* sp., described as a widespread parasite, could not be identified at the species level due to the lack of morphological data in studies. The two other *Leucocytozoon* lineages, lBAREGI252 and lBAREGI02, were detected in the South African *B. regulorum* in this study, and in *B. r. gibbericeps* in Rwanda. These lineages also could not be assigned to a specific *Leucocytozoon* species and exhibited significant genetic divergence (up to 4%) compared to other *Leucocytozoon* lineages available in the MalAvi and GenBank databases.

## Conclusion

This study advances the understanding of haemosporidian parasites in Gruidae birds, revealing significant molecular and morphological diversity among *Haemoproteus*, *Plasmodium*, and *Leucocytozoon* species. The re-description of *Haemoproteus balearicae* and its associated lineages highlights its broader host range and geographic distribution. Additionally, molecular analyses suggest cryptic speciation within *Haemoproteus*, emphasizing the need for taxonomic revision of *H. antigonis*. The detection of diverse *Plasmodium* and *Leucocytozoon* lineages, including the identification of the pCATUS05 lineage previously associated with *P. lutzi* group in South African cranes, suggests broad geographic and host ranges of these parasites. The lack of morphological data for many lineages highlights the critical need for integrative approaches to better characterize haemosporidian diversity and to assess its implications for crane health and conservation.

## Supplementary Files

*Supplementary Table 1*: Haemosporidian parasite prevalence based on PCR results, in SA Crane species and per site. *Supplementary Table 2*: *Haemoproteus*/*Plasmodium* and *Leucocytozoon* PCR prevalence among positive samples. The supplementary material of this article is available at https://www.parasite-journal.org/10.1051/parasite/2025059/olm.
