# Supplementary material for: Diversity of haemosporidian parasites in cranes: description of Haemoproteus balearicae and its phylogenetic position within the H. antigonis clade
Source: Parasite. 2025 Oct 13;32:66. doi: 10.1051/parasite/2025059 (PMC12520626; doi:10.1051/parasite/2025059)
Supplement: Supplementary file 1 — Supplementary Table 1: Haemosporidian parasite prevalence based on PCR results, in SA Crane species and per site. [file parasite-32-66-s1.pdf]

Supplementary Table 1. Haemosporidian parasite prevalence based on PCR results in South African Crane species and per site

Locality (No. of samples)	Blue Crane (*Anthropoides paradiseus*)	Grey Crowned Crane (*Balearica regulorum*)	Wattled Crane (*Bugeranus carunculatus*)	
**KwaZulu-Natal (KZN) n=2**	-	-	0/2	0%
**Eastern Cape (EC) n=40**	7/19 (36.8%)	12/21 (57.1%)	-	47.50%
**Gauteng Province (GP) n=105**	15/60 (25%)	19/38 (50%)	2/7 (28.6%)	34.30%
**TOTAL n=147**	22/79 (27.8%)	31/59 (52.5%)	2/9 (22.2%)	55/147 (37.4%)
