# Supplementary material for: Diversity of haemosporidian parasites in cranes: description of Haemoproteus balearicae and its phylogenetic position within the H. antigonis clade
Source: Parasite. 2025 Oct 13;32:66. doi: 10.1051/parasite/2025059 (PMC12520626; doi:10.1051/parasite/2025059)
Supplement: Supplementary file 2 — Supplementary Table 2: Haemoproteus/Plasmodium and Leucocytozoon PCR prevalence among positive samples. [file parasite-32-66-s2.pdf]

Supplementary Table 2. *Haemoproteus*/*Plasmodium* and *Leucocytozoon* PCR prevalence among positive samples

	**Blue crane (*Anthropoides paradiseus*) n=22**			**Grey crowned crane (*Balearica regulorum*) n=31**			**Wattled crane (*Bugeranus carunculatus*) n=2**		
**Locality (No. of samples)**	P/H	L	Mixed	P/H	L	Mixed	P/H	L	Mixed
**Eastern Cape (EC) n=19**	4	2	1	8	1	3	-	-	-
**Gauteng Province (GP) n=36**	13	2	0	14	2	3	2	-	-
**TOTAL n=55**	17 (77.3%)	4 (18.2%)	1 (4.5%)	22 (71%)	3 (9.7%)	6 (19.3%)	2 (100%)	-	-

P/H – *Plasmodium*/*Haemoproteus*; L – *Leucocytozoon*
